# Neuroprotective Effects of the Anti-cancer Drug Lapatinib Against Epileptic Seizures via Suppressing Glutathione Peroxidase 4-Dependent Ferroptosis

**DOI:** 10.3389/fphar.2020.601572

**Published:** 2020-12-10

**Authors:** Ji-Ning Jia, Xi-Xi Yin, Qin Li, Qi-Wen Guan, Nan Yang, Kang-Ni Chen, Hong-Hao Zhou, Xiao-Yuan Mao

**Affiliations:** ^1^ Department of Clinical Pharmacology, Xiangya Hospital, Central South University, Changsha, China; ^2^ Institute of Clinical Pharmacology, Central South University, Hunan Key Laboratory of Pharmacogenetics, Changsha, China; ^3^ Engineering Research Center of Applied Technology of Pharmacogenomics, Ministry of Education, Changsha, China; ^4^ National Clinical Research Center for Geriatric Disorders, Changsha, China; ^5^ Department of Pediatrics, Xiangya Hospital, Central South University, Changsha, China

**Keywords:** lapatinib, epileptic seizures, neuroprotection, ferroptosis, lipid peroxidation, glutathione peroxidase 4

## Abstract

Epilepsy is a complex neurological disorder characterized by recurrent and unprovoked seizures. Neuronal death process is implicated in the development of repetitive epileptic seizures. Therefore, cell death can be harnessed for ceasing seizures and epileptogenesis. Oxidative stress is regarded as a contributing factor of neuronal death activation and there is compelling evidence supporting antioxidants hold promise in abrogating seizure-related cell modality. Lapatinib, a well-known anti-cancer drug, has been traditionally reported to exert anti-tumor effect via modulating oxidative stress and a recent work illustrates the improvement of encephalomyelitis in rodent models after lapatinib treatment. However, whether lapatinib is beneficial for inhibiting neuronal death and epileptic seizure remains unknown. Here, we found that lapatinib remarkably prevented kainic acid (KA)-epileptic seizures in mice and ferroptosis, a newly defined cell death which is associated with oxidative stress, was involved in the neuroprotection of lapatinib. In the ferroptotic cell death model, lapatinib exerted neuroprotection via restoring glutathione peroxidase 4 (GPX4). Treatment with GPX4 inhibitor ras-selective lethal small molecule 3 (RSL3) abrogated its anti-ferroptotic potential. In a mouse model of KA-triggered seizure, it was also validated that lapatinib blocked GPX4-dependent ferroptosis. It is concluded that lapatinib has neuroprotective potential against epileptic seizures via suppressing GPX4-mediated ferroptosis.

## Introduction

Epilepsy is a relentless neurological disorder characterized by recurrent seizures which are manifestations of synchronized abnormal electrical activity (Loscher 2011; Feldman et al., 2018). Globally, it is estimated that nearly 70 million individuals, with an annual incidence of 80 per 100,000 population, suffer from epileptic damage (Moshe et al., 2015). Patients with epilepsy usually experience social prejudice, misunderstanding and unsuspected stress in life. Undoubtedly, this kind of disease has detrimental effects on people’s life quality.

The pathological mechanism of epilepsy is complex and multifactorial. Our previous publications have shown the evidence that neuronal death process is implicated in seizure generation (Mao et al., 2019b). Seizure-related cell death pathway activation is reported to result in hippocampal neuron loss and spatial memory impairment while abrogation of neuron death signaling ceases epileptic seizures and improves epilepsy-associated brain damage (Kotloski et al., 2002). Given that limited capacity of self-renewal in neurons (Sorrells et al., 2018), targeting neuronal death by neuroprotection holds promise in neurological disease therapy (Acharya et al., 2008; Neuhaus et al., 2017). For instance, resveratrol pretreatment potently reduces hippocampal neuron death in kainic acid (KA)-induced epileptic seizures (Wang et al., 2004). Nowadays, although more than one dozen of new anti-epileptic drugs have been introduced in the past 20 years, approximately one-third of patients fail to achieve complete remission (Wang and Chen, 2019). Therefore, it is of desperate need for development of novel drugs which counteract epileptic seizures and block epileptogenesis.

Lapatinib is a tyrosine kinase inhibitor which is approved for the treatment of breast cancer (Konecny et al., 2006). Recently, it has shown that lapatinib effectively improves autoimmune encephalomyelitis, a common central nervous system (CNS) dysfunction (Akama-Garren et al., 2015). Additionally, owing to low molecular weight and lipophilic properties, lapatinib is considered to efficiently penetrate the blood-brain barrier in the CNS (Gori et al., 2014), which also supports the notion that it has good therapeutic potential in the CNS. Our current work aimed to decipher whether lapatinib protects against epileptic seizures and the potential molecular mechanism.

Oxidative stress is a critical factor contributing to neuronal death caused by severe and repetitive seizures (Ferriero, 2005). Previous experimental results have unequivocally proved that epileptic seizures-mediated oxidative stress activates neuronal death (Liang et al., 2000; Mao et al., 2019a). Brain is particularly vulnerable to oxidative damage due to higher oxygen consumption (about 20%) and lower antioxidant capacity than other organs (Cobley et al., 2018). Additionally, given that abundance of polyunsaturated fatty acids, which are prone to oxidized lipids, in the neuronal membrane (Cobley et al., 2018), lipid peroxidation is a dominant form of oxidative stress. Recently, ferroptosis, a newly defined cell death mode, which is featured by accumulation of iron-dependent lipid peroxidation, has been identified by Stockwell laboratory (Dixon et al., 2012). It has been extensively reported to be involved in diverse pathological conditions including Alzheimer’s disease (AD), Parkinson’s disease (PD), cancer, ischemia-reperfusion injury and so on (Xie et al., 2016; Guiney et al., 2017). Interestingly, Ye research group and our prior work also reveals contribution of ferroptosis to seizure generation in various epileptic rodent models (Mao et al., 2019a; Ye et al., 2019). Pharmacological inhibition of ferroptosis process by an herb baicalein ameliorates seizure behavior (Li et al., 2019). Due to the previous results that lapatinib regulates oxidative stress in cancer and can cure encephalomyelitis (Aird et al., 2012; Ma et al., 2017), it is rational to speculate that lapatinib likely eradicates oxidative stress-associated ferroptosis and protects against epileptic seizures.

## Materials and Methods

### Chemicals and Reagents

Erastin, lapatinib, ferrostatin-1 (Fer-1), liproxstatin-1 (Lip-1) and Ras-selective lethal small molecule 3 (RSL3) were obtained from Selleck Chemicals (Houston, TX, United States). Glutamate (Glu), propidium iodide (PI), Hoechst 33342, KA and deferoxamine (DFO) were obtained from Sigma-Aldrich (St. Louis, MO, United States). Dulbecco’s modified Eagle’s medium (DMEM), Hank’s Balanced Salt Solution (HBSS) and fetal bovine serum (FBS) were obtained from GIBCO (Grand Island, NY, United States). MDA Assay Kit and GSH and GSSG Assay Kit were purchased from Beyotime (Shanghai, China).

### Preparation of KA-Induced Seizure Model

Male C57BL/6J mice (6–8 weeks of age, weighing 18–22 g) were obtained from the Animal Unit of Central South University. All C57BL/6J mice were housed in an indoor environment with a 12 h light/12 h dark cycle, 24 ± 2°C and other specific laboratory conditions, with free access to food and water. Experimental protocols for all animals were approved by the Ethical Committee of Xiangya Hospital Central South University. After anesthetization by intraperitoneal injection of 10% chloral hydrate (v/w), the mice were fixed on a stereotactic instrument and stereotactically injected with KA (250 ng/μl) into the hippocampus. KA (1 µl) was injected slowly for 5 min and positioned in the hippocampus (AP—2.0 mm, ML—1.3 mm, V—1.2 mm). After injection, the needle was left in place for additional 10 min to avoid drug reflux. The mice were randomly divided into six experimental groups: 1) sham operation group that received 1 µl PBS injection (5 animals); 2) mice were pretreated p. o. for 21 days on a twice-daily schedule with 100 mg/kg lapatinib alone before PBS administration (5 animals); 3) KA-treated group was injected KA (5 animals); 4) and 5) lapatinib groups were received with 50 mg/kg (5 animals) and 100 mg/kg (5 animals) lapatinib for 21 days before KA treatment, respectively; 6) this group was given i. p. for 14 days with ferroptosis inhibitor (3 mg/kg Fer-1) before KA administration.

### Behavioral Observation

The behavioral changes of epileptic mice were observed successively for 90 min after injection of KA. The symptoms of epileptic seizures were assessed by a scoring system which is defined by Racine (1972). The standards of Racine stages were described as follows: stage 0, no convulsions and other responses; stage 1, facial and whisker rhythmic twitching; stage 2, head bobbing and circling; stage 3, myoclonic and spasm in multiple limbs; stage 4, uncontrolled rearing and falling; stage 5, general tonic-clonic seizures with running and jumping; stage 6, death. If the symptoms of the third or higher stage were observed, the animals were considered to have seizures.

### Nissl Staining

After anaesthetization with 10% (v/w) of chloral hydrate, the animals experienced transcardial perfusion with physical saline and 4% paraformaldehyde. The brains were postfixed in 4% paraformaldehyde for 2 days. Brain tissue sections (10 μm) including the whole hippocampus were immersed in cresyl violet (C0117, Beyotime Biotechnology, China) for 10 min at room temperature. Following dehydration in graded alcohol, the slides were coverslipped with neutral balsam and observed with a light microscope (Leica Application Suite, 4.9.0, Weztlar, Germany).

### Electroencephalogram (EEG) Recording

EEG was recorded by two bipolar electrodes punctured scalp. After anesthetization with 10% chloral hydrate (v/w), the animals were fixed carefully on a stereotactic instrument. A guide cannula (62004, RWD Life Science) and a bipolar electrode were vertically implanted into the hippocampal CA1 subregion (AP − 2.0 mm; ML − 1.3 mm; V—1.2 mm) and CA3 (AP − 2.9 mm; ML—3.0 mm; V—3.0 mm), respectively, on the basis of the mouse brain atlas. After 1-week recovery, the EEG baseline was obtained via recording for 10 min prior to hippocampal KA infusion. Then, an injection tube (62204, RWD Life Science) was inserted into the right hippocampus through the guide cannula with a depth of 1 mm for KA (250 ng/μl) injection. Following slow injection of KA (1 µl) for 5 min, the injection tube was left in the hippocampus for 5 min to avoid reflux. At this moment, EEG recording was continued to be captured. Seizure events were defined as threefold-baseline high amplitude discharges with spike frequency greater than 3 Hz.

### Cell Culture

Immortalized mouse hippocampal cell line HT22 was cultivated in high-glucose DMEM (C11995500BT, Gibco, United States) containing 10% FBS (10270-106, Gibco, United States), 100 U/ml penicillin and 100 μg/ml streptomycin that maintained in a 5% CO_2_ incubator at 37°C.

### Cell Death Assay

HT22 cells were cultured in a 24-well plate with a density of 10%. Different concentrations of lapatinib (S1028, Selleck, United States) were pretreated to cells at various time points, and then HT22 cells were exposed to Glu (G8415, Sigma, United States) or erastin (S7242, Selleck, United States). After incubation of Glu or erastin for 8 h, PI and Hoechst 33342 at the concentration of 5 μg/ml were treated for 10 min. Cell death rate was measured by PI (+)/Hoechst (+). The percentage of cell death was performed according to the previous publications (Mi et al., 2012).

### Real-Time Quantitative PCR

After drug treatment at the indicated time, total RNA from cells and hippocampal tissues were extracted using TRIzol reagent (Invitrogen, United States) following the manufacturer’s procedures. One microgram of total RNA for each sample was reversely transcribed into cDNA by a commercial kit (RR047A, Takara Bio, Japan) according to the manufacturer’s protocol. Real-time PCR was carried out using SYBR Green PCR Master Mix (RR091A, Takara, Japan) and the quantitative analysis was performed using LightCycler Roche 480 qPCR instrument. The conditions for PCR were as follows: 30 s hot-start at 95°C followed by 40 cycles of 5 s at 95°C, 30 s at 55°C and 30 s at 72°C; 30 s melting curve at 95°C. All samples were tested in triplicate, and expression levels were normalized to β-actin gene expression levels. Primer sequences used for real-time quantitative PCR were displayed as follows: prostaglandin endoperoxide synthase 2 (PTGS2) forward: 5′-GGGAGTCTGGAACA.

TTGTGAA-3′, reverse: 5′-GTG​CAC​ATT​GTA​AGT​AGG​TGG​ACT-3′; β-actin forward: 5′-GTG​ACG​TTG​ACA​TCC​GTA​AAG​A-3′, reverse: 5′-GCC​GGA​CTC​ATC​GTA​CTC​C-3′

### Immunofluorescence

HT22 cells were cultivated in 35 mm dishes at a density of 15%. After drug treatments, the cells were fixed with 4% paraformaldehyde for 20 min and infiltrated with 0.2% Triton X-100 for 15 min, followed by blocking in donkey serum for 30 min and incubating overnight at 4°C with primary antibody (GPX4, rabbit, ab125066, 1:200, Abcam) in PBS. On the second day, after three washes with PBS, cells were incubated with donkey anti-rabbit IgG (A21206, 1:100, Thermo Fisher Scientific, United States). Finally, the cell nuclei were stained with DAPI (S2110, Solarbio, China) for 10 min. Fluorescent results were analyzed under a laser-scanning confocal microscope (Nikon, Japan).

### Western Blot Analysis

After drug treatment, HT22 cells or hippocampal tissues were harvested and lysed in RIPA buffer supplemented with protease and phosphatase inhibitor. Cell and tissue lysates were sonicated for 30 s. The supernatants were retained and subsequently quantified using BCA protein assay kit (P0006, Beyotime Biotechnology, China). In brief, 20 μg proteins for each sample were subjected to sodium dodecyl sulfate–polyacrylamide gel electrophoresis and then electrophoretically transferred to polyvinylidene fluoride membranes. After blocking with TBST containing 5% nonfat milk for 1 h, the membranes were incubated with GPX4 (rabbit, 22 kDa, ab125066, 1:5,000, Abcam), 4-hydroxynonenal (4-HNE) (rabbit, 17–76 kDa, ab46545, 1:2,500, Abcam), acyl-CoA synthetase long-chain family member 4 (ACSL4) (mouse, 75 kDa, sc-365230, 1:1,000, Santa Cruz), solute carrier family 7 member 11 (SLC7A11) (rabbit, 55 kDa, ab175186, 1:5,000, Abcam), Anti-5 Lipoxygenase (5-LOX) (rabbit, 78 kDa, ab169755, 1:1,000, Abcam) and β-actin (mouse, 43 kDa, A5441, 1:5,000, Sigma) overnight at 4°C. The next day, following three washes in TBST, the membranes were incubated with horseradish peroxidase (HRP)-conjugated anti-rabbit IgG (rabbit, A9169, 1:10,000, Sigma) or anti-mouse IgG (mouse, A9044, 1:10,000, Sigma) at room temperature for 1 h. Immunoreactivity was assessed using the ChemiDoc XRS + imaging system (Bio-Rad, United States). The protein band intensity was quantified by using ImageJ software (NIH, United States). The protein expression levels were normalized to β-actin.

### Measurement of Lipid Reactive Oxygen Species

HT22 cells were seeded in six well plates at the density of 15% and treated at indicated time points. Cells were washed twice with PBS, trypsinized, and then incubated with 500 μl HBSS (C14175500BT, Gibco, United States) supplemented with 2 μM C11-BODIPY 581/591 (D3861, Thermo Fisher, United States) at 37°C for 15 min. Then, the cells were washed twice with 500 μl HBSS, centrifuged for 5 min at 3,000 × *g* and re-suspended in 200 μl PBS. Finally, the fluorescence intensities were detected by flow cytometry and the data were analyzed using FlowJo software.

### Measurement of Malondialdehyde Level

The degree of lipid peroxidation can be analyzed by quantification of MDA. MDA content in HT22 cells was tested using a specific colorimetric kit (S0131, Beyotime Biotechnology, China) according to the manufacturer’s instructions. Briefly, MDA can react with thiobarbituric acid (TBA) to generate the MDA-TBA adduct which can be measured spectrophotometrically at 532 nm.

### Measurement of Glutathione Level

Assessment of GSH level in HT22 cells was detected using a GSH and GSSG assay kit (S0053, Beyotime Biotechnology, China) according to the manufacturer’s protocol. The absorbance was assayed at 412 nm with a microplate reader.

### Statistical Analysis

Results were presented as the mean ± SEM. Statistical significance was evaluated by one-way analysis of ANOVA with Tukey’s post hoc test among three groups using GraphPad Prism 5.0 software. Statistical differences with *p* values less than 0.05 were considered significant.

## Results

### Lapatinib Protects Mice Against KA-Induced Seizures

**FIGURE 1:**
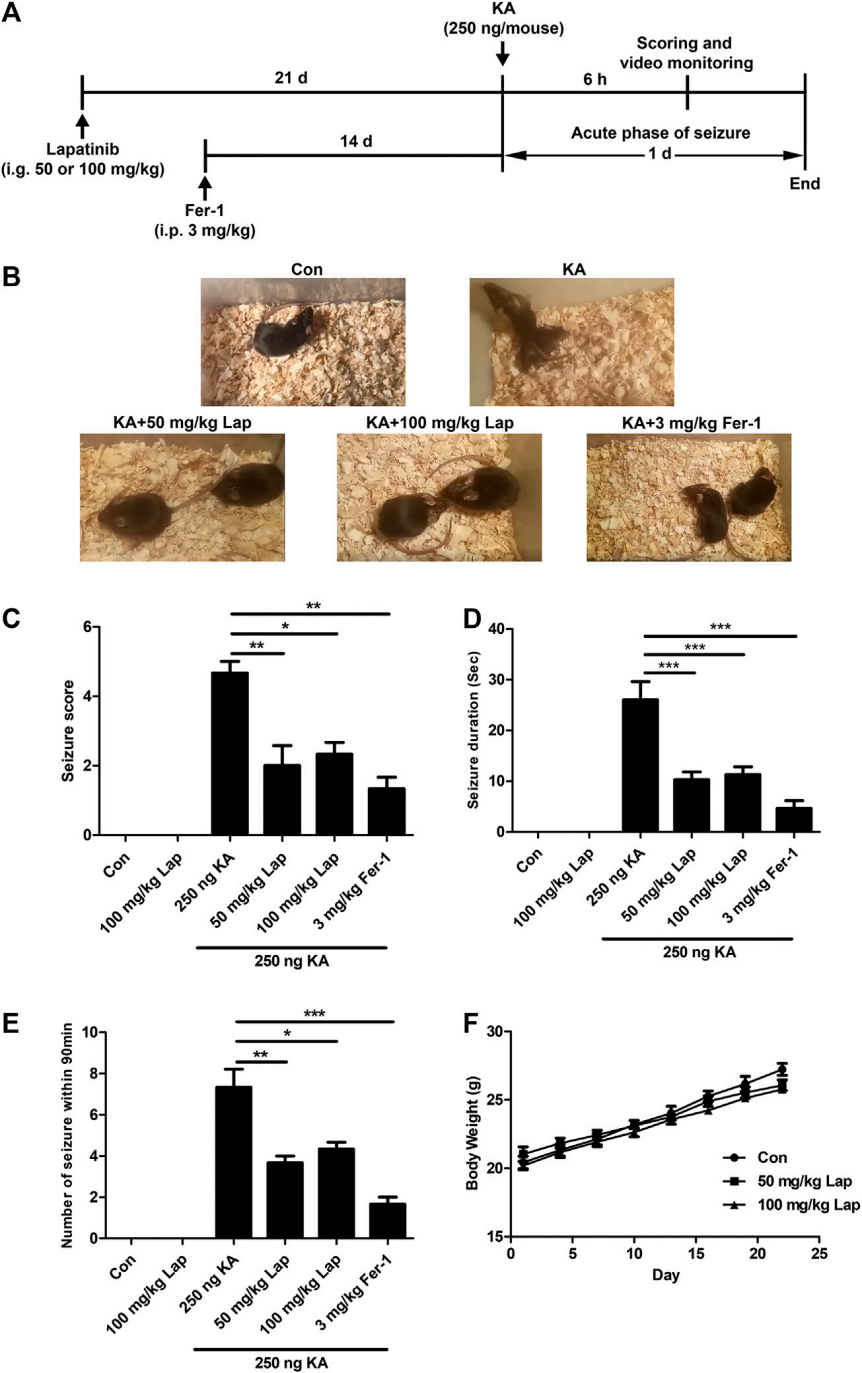
Lapatinib protects mice against KA-induced seizures **(A)** Experimental design **(B)** Representative images from different groups after pretreatment with lapatinib (Lap) or ferrostatin-1 (Fer-1) in KA-induced epileptic seizures **(C–E)** Effects of Lap or Fer-1 on seizure score, number of seizures and average seizure duration **(F)** Effects of Lap alone on body weight. All results were shown as the mean ± SEM (*n* = 5), *p < 0.05, **p < 0.01 and ***p < 0.001.

Initially, we examined whether lapatinib could exert neuroprotective effect and improve seizure behavior in a mouse model induced by KA. Figure 1A showed the experimental timeline. The rodent seizure model was prepared by injecting 1 μl 250 ng/μl kA into the hippocampus. The successful preparation of seizure model was evaluated by the appearance of obvious symptoms such as rollover and generalized ankylosing seizure. After treatment with lapatinib with different doses (50 or 100 mg/kg), seizure behavior was recorded within 90 min (Figure 1B and Supplementary Video). As shown in Figures 1C–E, lower seizure score (ANOVA, F (3,16) = 12.61, *p* = 0.02), shorter seizure duration (ANOVA, F (3,16) = 49.66, *p* < 0.001) and smaller number of seizures within 90 min (ANOVA, F (3,16) = 19.83, *p* < 0.001) were observed in a mouse model of KA-induced epileptic seizures when treatment with lapatinib. It was noted that lapatinib at the dose of 100 mg/kg exhibited more evident protection against seizure phenotype than that by the dose of 50 and 100 mg/kg lapatinib had no significant effect on weight gain, indicating a good tolerance of lapatinib (Figure 1F). Taken together, these results indicate that lapatinib has a neuroprotective effect in epileptic seizures.

Lapatinib alleviates seizure-induced hippocampal damage and electrical activity in KA-treated mice.

**FIGURE 2:**
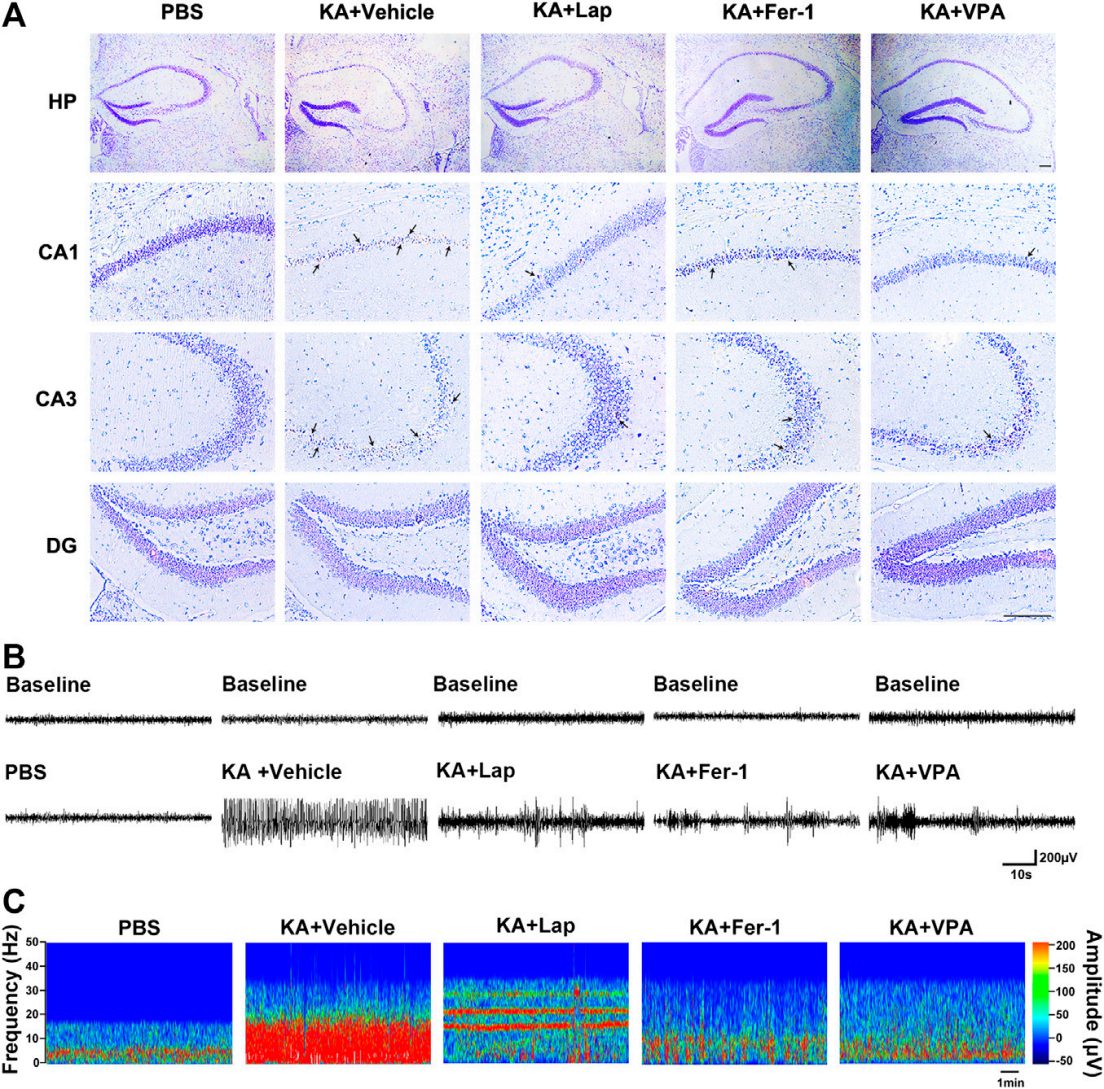
Lapatinib alleviates seizure-induced hippocampal damage and electrical activity in KA-treated mice **(A)** Nissl staining results displaying hippocampal subregions including CA1, CA3 and DG in KA-induced seizures following treatment with lapatinib (Lap), ferrostatin-1 (Fer-1), or valproate (VPA). Scale bar: 50 μm **(B,C)** Representative electroencephalogram recordings and power spectrum density within 10 min are shown in different groups which are the same as **(A)**.

We further analyzed seizure-induced brain damage in KA-treated mice when pretreatment with lapatinib via Nissl staining. As mentioned above, lapatinib by the dose of 50 mg/kg exerted potent improvement of seizure severity, which is comparable with treatment at the dose of 100 mg/kg. Therefore, the dose of 50 mg/kg was selected to explore the effect of lapatinib on seizure-induced hippocampal impairment in mice via Nissl staining. As shown in Figure 2A, the seizure mice of KA induction exhibited massive neuronal death in hippocampus, especially CA1 and CA3 subregions. However, treatment with lapatinib significantly improved neuronal viability, which is similar to the responses shown in ferroptosis inhibitor Fer-1 and the existing anti-seizure agent VPA.

In addition, we also carried out EEG recording to validate the neuroprotective potential of lapatinib against epileptic seizures. The drug design was similar to that in Nissl staining experiment. It was noteworthy that increased electrical activity was observed in KA-treated mice. Treatment with lapatinib, Fer-1 or VPA remarkably suppressed abnormal brain discharge (Figures 2B,C).

Taken together, these data further confirm that lapatinib has neuroprotective effect against epileptic seizures.

### Lapatinib Suppresses Ferroptosis in KA-Induced Epileptic Mice

**FIGURE 3:**
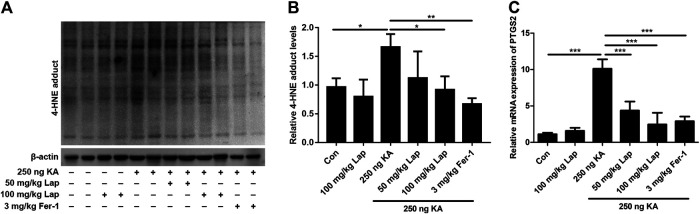
Lapatinib suppresses ferroptosis in KA-induced epileptic mice **(A,B)** Protein expressions of 4-HNE in hippocampus tissue samples of KA-induced seizure model with different doses of lapatinib (Lap) or Fer-1 pretreatment **(C)** RT-qPCR analysis of PTGS2 mRNA expression pretreated with lapatinib or Fer-1 in epileptic mice of KA injection. All results were shown as the mean ± SEM (*n* = 5), *p < 0.05, **p < 0.01, and ***p < 0.001.

To further investigate the potential molecular mechanism by which lapatinib exerted neuroprotection, the alterations of ferroptosis-related indices including 4-HNE (a common by-product of lipid peroxidation) (Hu et al., 2020) and PTGS2 (a potential molecular marker of ferroptosis) (Jiang, 2015) were detected in our present investigation. 4-HNE was the protein adducts that correspond to the dominant bands including 76, 52, and 17 KDa. As shown in Figures 3A,B, KA injection resulted in the upregulation of 4-HNE levels and this phenomenon was reversed after pretreatment with lapatinib and Fer-1 (ANOVA, F (5,24) = 5.219, *p* = 0.009). PTGS2 mRNA was also dramatically reduced in the seizure model after pretreatment with lapatinib or Fer-1 compared with KA treatment alone (ANOVA, F (5,24) = 62.07, *p* < 0.001) (Figure 3C). Consistent with our present work, the level of 4-HNE and PTGS2 mRNA were observed to be remarkably elevated in seizure models (Mao et al., 2019a). Collectively, these results indicate that lapatinib’s neuroprotection may be closely related to inhibition of ferroptosis.

### Lapatinib Exerts Neuroprotection Against Glu- or Erastin-Induced Cell Death in HT22 Neurons

To further explore the mechanism on how ferroptosis affects lapatinib’s neuroprotection against KA-induced epileptic seizures, we prepared a ferroptotic cell death model induced by Glu or erastin (a specific ferroptotic inducer). HT22 neuronal cell line is regarded as an ideal model for investigating oxidative stress due to deficiency of N-methyl-d-aspartate (NMDA) receptor (Jin et al., 2014).

**FIGURE 4:**
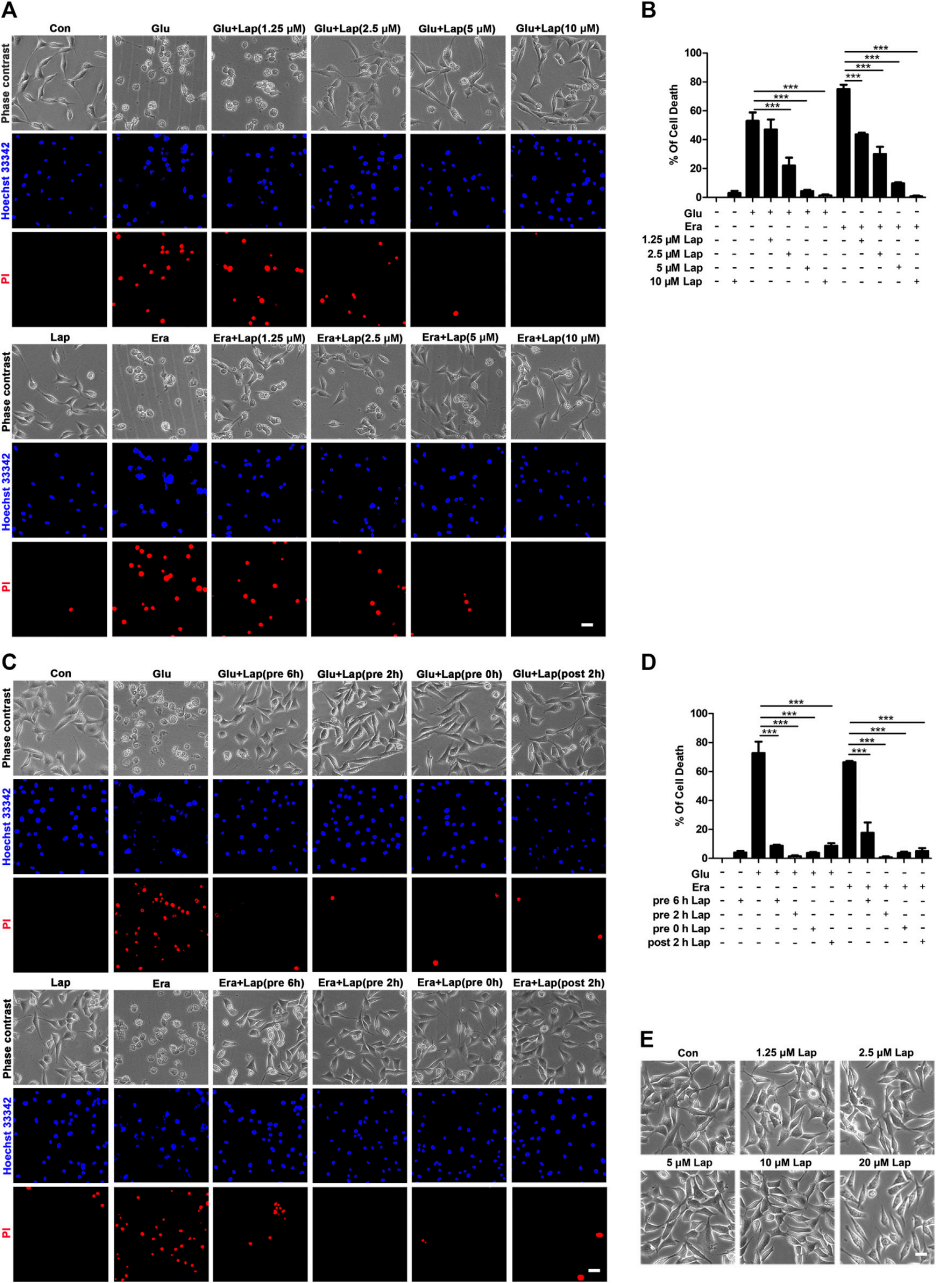
Lapatinib exerts neuroprotection against Glu- or erastin-induced cell death in HT22 neuronal cell line **(A)** indicated HT22 cells were treated with glutamate (Glu) (5 mM) or erastin (Era) (0.5 μM) for 8 h. Cell morphology was observed by phase-contrast microscopy after pretreatment with lapatinib (Lap) at different concentrations (1.25, 2.5, 5, and 10 μM) for 2 h. Scale bar: 200 μm **(B)** HT22 cells were pre-incubated with Lap (1.25, 2.5, 5, and 10 μM) for 2 h, followed with Glu or erastin treatment. Cell death rate was measured by PI (+)/Hoechst (+) **(C,D)** Lapatinib (10 μM) was exposed with Glu or Era challenge at different periods of time (before 6 h, 2 h, simultaneously or post 2 h). The percentage of cell death rate was measured by PI (+)/Hoechst (+). Scale bar: 200 μm **(E)** Effects of different concentrations of Lap alone on HT22 cells. Scale bar: 200 μm. All results were shown as the mean ± SEM from three independent experiments, ***p < 0.001.

There is substantial evidence showing that ferroptosis occurs in Glu-induced HT22 toxicity (Kang et al., 2014). Simultaneously, we also used the specific ferroptosis model in HT22 cells induced by erastin as a positive control. We detected the effects of lapatinib on ferroptotic cell death in HT22 cells at different doses for different time points. It was found that pretreatment with lapatinib at indicated range of concentrations (1.25–10 μM) significantly diminished HT22 cell death induced by Glu or erastin, and its protective effects reached the peak at 10 μM (ANOVA, F (10,22) = 45.46, *p* < 0.001) (Figures 4A,B). Therefore, 10 μM was selected in the subsequent experiments. In order to ascertain the optimal time point, lapatinib at the concentration of 10 μM was treated in the different time points: pretreatment for 6 h (pre 6 h), pretreatment for 2 h (pre 2 h), co-treatment with Glu or erastin (pre 0 h) and posttreatment for 2 h (post 2 h). It was noteworthy that pretreatment of 10 μM lapatinib for 2 h exerted the most protective effect on Glu- or erastin-induced ferroptosis (ANOVA, F (10,22) = 60.77, *p* < 0.001) (Figures 4C,D). Treatment with lapatinib alone had no toxic effect on HT22 cells (Figure 4E). These findings reveal neuroprotection of lapatinib against Glu-induced neuronal death.

### Lapatinib Prevents Glu- or Erastin-Induced Neuronal Death Possibly by Suppressing Ferroptosis

**FIGURE 5:**
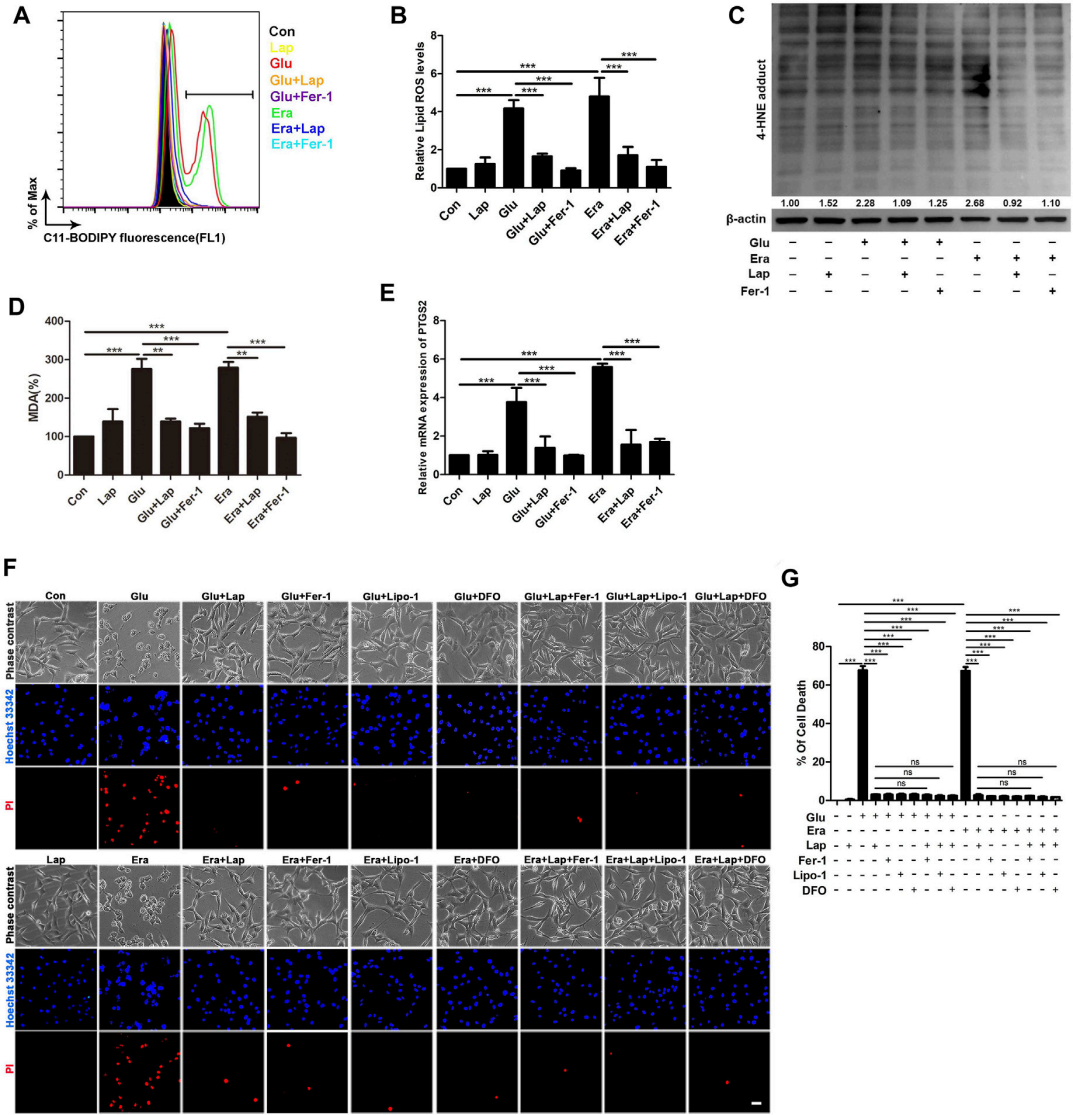
Lapatinib prevents Glu- or erastin-induced neuronal death possibly by suppressing ferroptosis (**A–D)** Detection of lipid ROS, 4-HNE and MDA content in the glutamate (Glu)- or erastin (Era)-induced HT22 cell death model following lapatinib (Lap) (10 μM) and ferrostatin-1 (Fer-1) (12.5 μM) pretreatment for 2 h **(E)** RT-qPCR analysis of PTGS2 mRNA expression pretreated with or without Lap (10 μM) and Fer-1 (12.5 μM) in HT22 cells induced by Glu or Era **(F,G)** Comparisons of combination of Lap and ferroptosis inhibitors and Lap alone in HT22 cells following Glu or Era challenge when pretreatment with Lap (10 μM), Fer-1 (12.5 μM), liproxstatin-1 (Lip-1) (1 μM) and deferoxamine (DFO) (50 μM) pretreatment for 2 h. Scale bar: 200 μm. All results were presented as the mean ± SEM from three independent experiments, ns indicates no statistical significance. **p < 0.01 and ***p < 0.001.

In order to further explore whether the neuroprotective effect of lapatinib was correlated with inhibition of ferroptosis, lipid peroxidation (the feature of ferroptosis) was detected in our present work. As shown in Figures 5A,B, an evident decrease of lipid ROS accumulation was observed in Glu- or erastin-induced ferroptosis after lapatinib pretreatment (ANOVA, F (7,16) = 34.46, *p* < 0.001). In a ferroptotic cell death model in HT22, lipid ROS content was significantly increased compared with control group (Jelinek et al., 2018). We detected overwhelming lipid ROS after Glu and erastin exposure in HT22 cells. And lapatinib also remarkably suppressed other ferroptotic indices, including 4-HNE levels (Figure 5C), MDA (a product of lipid metabolism) (ANOVA, F (7,8) = 35.72, *p* < 0.001) (Figure 5D), and PTGS2 mRNA (ANOVA, F (7,40) = 114.7, *p* < 0.001) (Figure 5E). Additionally, combinational treatment with lapatinib and ferroptosis inhibitor (Fer-1, Lip-1 or DFO) did not induce more serious toxicity in HT22 cells following Glu or erastin exposure than lapatinib alone (ANOVA, F (16,34) = 773, *p* < 0.001) (Figures 5F,G). It implicates that lapatinib exerts protection against neuronal impairment possibly by modulating ferroptosis.

### Lapatinib Inhibits Neuronal Ferroptosis by Maintaining Glutathione Peroxidase 4

**FIGURE 6:**
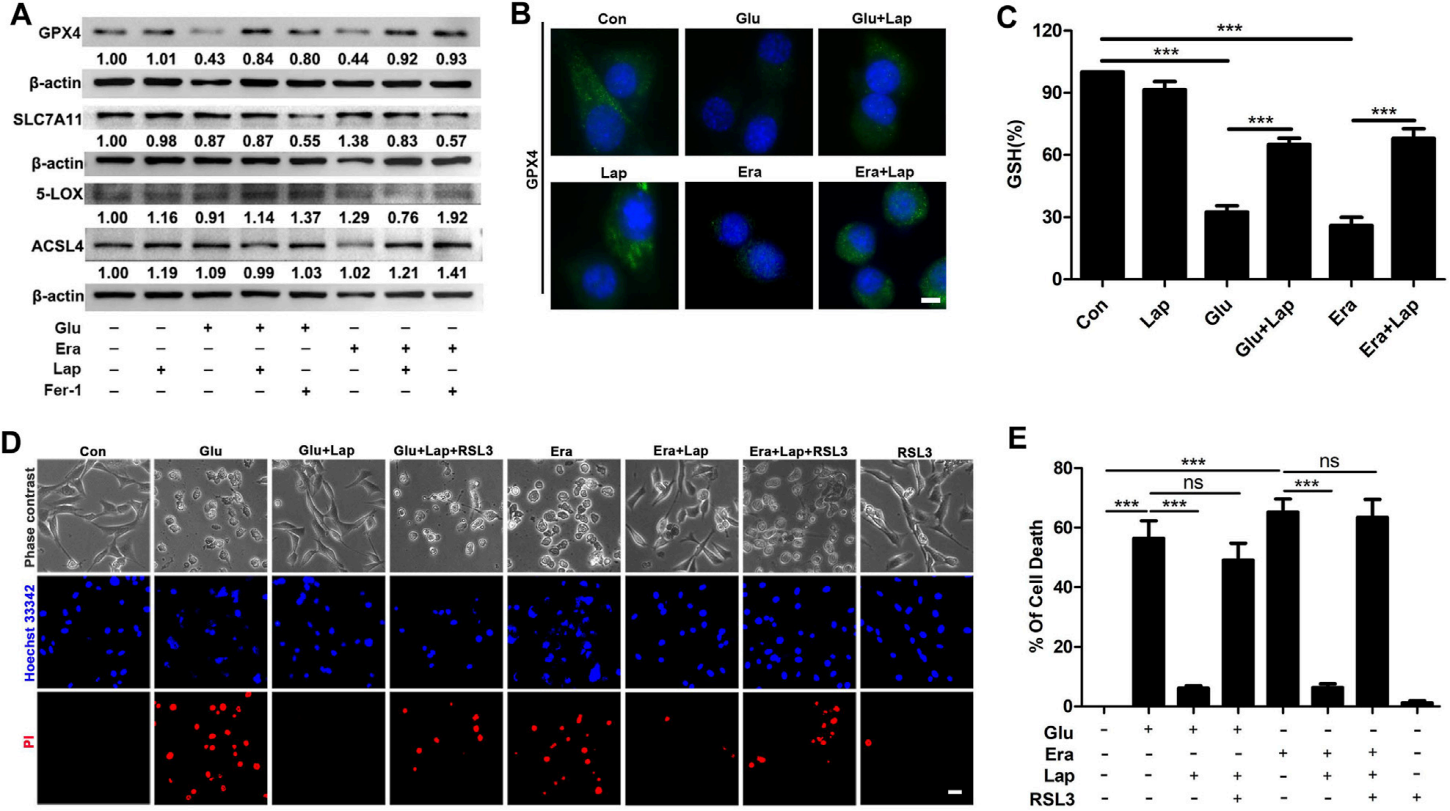
Lapatinib inhibits neuronal ferroptosis by blocking the downregulation of GPX4 **(A)** Effects of lapatinib (Lap) or ferrostatin-1 (Fer-1) on the expressions of ferroptosis-related proteins including glutathione peroxidase 4 (GPX4), solute carrier family 7 member 11 (SLC7A11), 5-Lipoxygenase (5-LOX) or acyl-CoA synthetase 4 (ACSL4) in HT22 cell death model induced by glutamate (Glu) or erastin (Era). The value below the band is obtained via ImageJ analysis **(B)** Immunofluorescence analysis of GPX4 expression after pretreatment for 2 h with Lap (10 μM), followed by exposure to Glu or Era for another 8 h in HT22 cells. Scale bar: 50 μm **(C)** GSH level was assessed following Lap (10 μM) pretreatment for 2 h **(D,E)** Effects of GPX4 inhibition by ras-selective lethal small molecule 3 (RSL3) (1 nM) on the neuroprotection of Lap (10 μM) against Glu- or erastin-induced cell death in HT22 cells. Scale bar: 200 μm. All results were shown as the mean ± SEM from three independent experiments, ns means the difference is not statistically significant, ***p < 0.001.

Next, we probed the molecular mechanism of lapatinib’s neuroprotection against neuronal ferroptosis. We examined diverse factors including GPX4 (Ingold et al., 2018), SLC7A11 (Sehm et al., 2016), 5-LOX (Sun et al., 2019) and ACSL4 (Doll et al., 2017) (Figure 6A) which were associated with lipid peroxidation. We found the most evident maintenance of GPX4 in the Glu- or erastin-induced HT22 cell injury model after pretreatment with lapatinib. Meanwhile, we also detected the expression level of GPX4 in the Glu- or erastin-induced cell damage model by immunofluorescence method. Compared with the KA-treated group, the immunofluorescence signal of GPX4 was significantly maintained in the lapatinib pretreatment group (Figure 6B). Additionally, GPX4 transforms toxic lipid peroxide into nontoxic lipid alcohols by using GSH as an auxiliary factor in lipid peroxidation-dependent ferroptosis (Imai et al., 2017). As shown in Figure 6C, in HT22 cells following Glu or erastin treatment, lapatinib inhibited the decrease of intracellular GSH levels (ANOVA, F (5,16) = 285.1, *p* < 0.001), which inversely correlate with lipid peroxidation (Ayala et al., 2014). GSH is the substrate for GPX4 to exert an antioxidant effect. Consistent with previous reports, cellular GSH levels were significantly decreased in HT22 cells after exposure to Glu or erastin (Liu et al., 2015). Given that consumption of GPX4 *in vitro* can be reversed by lapatinib, we subsequently explored whether treatment with a sublethal dose of RSL3, which inhibits GPX4 but did not cause cell death (Figures 6D,E), could reverse the protective effect of lapatinib (ANOVA, F (6,14) = 48.25, *p* < 0.001). It was noted that treatment with RSL3 abrogated neuroprotection of lapatinib against neuronal ferroptosis. Overall, these data strongly support that lapatinib exerts an inhibitory role on neuronal ferroptosis by blocking the downregulation of GPX4.

### GPX4 is Involved in the Inhibition of Ferroptosis of Lapatinib in KA-Epileptic Seizures

**FIGURE 7:**
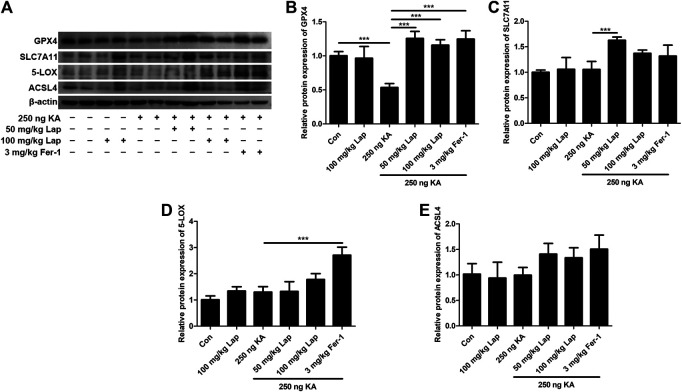
GPX4 is involved in the inhibition of ferroptosis of lapatinib in KA-epileptic seizures **(A)** Western blot showing expression of glutathione peroxidase 4 (GPX4), solute carrier family 7 member 11 (SLC7A11), 5-Lipoxygenase (5-LOX) or acyl-CoA synthetase 4 (ACSL4) in hippocampus tissue samples of KA-induced epileptic mice with different doses of Lap and Fer-1 pretreatment **(B–E)** Quantitative analysis of GPX4, SLC7A11, 5-LOX and ACSL4. All results were shown as the mean ± SEM (*n* = 5), ***p < 0.001.

Subsequently, we validated the inhibitory role of lapatinib on neuronal ferroptosis in a mouse model of KA-triggered epileptic seizures. Consistent with *in vitro* results, GPX4 expression level in the hippocampus was significantly increased after pretreatment with lapatinib compared to the KA-treated group (ANOVA, F (5,24) = 25.25, *p* < 0.001) (Figures 7A,B). Recent studies have consistently found that GPX4 is decreased in epileptic rodent models (Mao et al., 2019a; Li et al., 2019). Other identified ferroptosis targets including SLC7A11 (ANOVA, F (5,24) = 10.68, *p* < 0.001), 5-LOX (ANOVA, F (5,24) = 23.53, *p* < 0.001) and ACSL4 (ANOVA, F (5,24) = 4.521, *p* = 0.008) were slightly or hardly affected in KA-induced epileptic seizures when treatment with lapatinib (Figures 7A,C–E). Collectively, these results indicate that lapatinib protects mice against ferroptosis in KA-triggered epileptic seizures via halting GPX4-dependent ferroptosis.

## Discussion

The major finding of our current work illustrated that lapatinib exerted neuroprotection against epileptic seizures. Furthermore, it was found that the neuroprotective potential of lapatinib was at least in part related to inhibition of GPX4-dependent neuronal ferroptosis.

Recently, drug repurposing (also known as drug repositioning) is a promising strategy of drug development and has attracted considerable attention (Oprea and Mestres, 2012). Generally, drug repurposing refers to dig out the new usage of clinically approved drugs and dosage forms for existing drugs or drug candidates. In order to reduce the time period in the process of drug development, it is rational to develop antiepileptic drugs by drug repositioning (Brueggeman et al., 2019). Compared with the development of new drugs, this approach has the advantage of tolerable pharmacokinetic, pharmacodynamic and toxicological properties (Nosengo, 2016). Here, we focus on the tyrosine kinase inhibitors which traditionally display potent anti-cancer effect. A growing number of studies have demonstrated that the compounds which inhibit tyrosine kinase not only kill cancer cells but also show promise in the non-neoplastic field. For example, asthmatic responses and autoimmune arthritis could be effectively cured by imatinib (Berlin and Lukacs 2005; Paniagua et al., 2006). In the research of neurological disease therapy, sunitinib, a traditional anti-cancer drug, provided an attractive therapeutic strategy for protecting HIV-related neurotoxicity (Wrasidlo et al., 2014). Several studies have also delineated that imatinib ameliorates AD by inhibition amyloid-β formation (He et al., 2010). Besides, the therapeutic effect of lapatinib is observed in experimental autoimmune encephalomyelitis (Akama-Garren et al., 2015). These findings highlight great therapeutic potential in CNS pathology.

Our results showed that lapatinib’s neuroprotection was achieved by suppressing ferroptosis in neurons. Ferroptosis, a newly discovered form of regulated necrosis, is characterized by iron accumulation in cellular and excessive occurrence of lipid peroxidation (Dixon, 2012). Numerous reports have established that ferroptosis is closely associated with multiple pathological processes, such as cancer, neurotoxicity, acute kidney failure, liver injury and so on (Xie et al., 2016). Our previous publications have demonstrated that ferroptosis appears in iron chloride-induced epileptic seizure models and treatment with ferroptosis inhibitor Lip-1 significantly improves behavioral seizures, indicating therapeutic value of ferroptosis inhibition on epileptic seizures (Li et al., 2019). Similarly, in the KA- or pilocarpine-induced seizure model, treatment with ferroptosis inhibitor Fer-1 also remarkably counteracts epileptic seizures (Mao et al., 2019a; Ye et al., 2019). In our current study, we uncovered that lapatinib protected against epileptic seizures via suppressing ferroptosis in murine models by KA injection. In contrast, previous investigations indicated that lapatinib exerted anti-cancer effects via inducing ferroptosis (Ma et al., 2016; Ma et al., 2017). The discrepancy may be attributable to different cell contexts (Mao et al., 2018). Our work further proved that GPX4, a vital antioxidant enzyme (Fricker et al., 2018), was involved in the neuroprotective effects of lapatinib. GPX4 plays a crucial role in inhibiting the accumulation of lipid ROS and scavenges lipid peroxidation under oxidative stress. In contrast, a specific inhibitor of GPX4 can initiate ferroptosis (Yang et al., 2014). In our previous work, we found that RSL3 at a concentration of 12.5 nM for 8 h exerted potent damage on HT22 cells. However, we chose the sublethal dose of RSL3 (1 nM) to act on HT22 cells. It is more convincing to reverse the protective effect of lapatinib on ferroptosis caused by Glu or erastin, while RSL3 at the concentration of 1 nM does not cause cell death. GPX4 is a major regulator of ferroptosis. And lots of investigations have shown that GPX4 is related to the development process of human diseases (Friedmann Angeli et al., 2014; Matsushita et al., 2015). Mice lacking GPX4 in the brain are vulnerable to immune dysfunction and tissue impairment. In fact, motor neurons are especially sensitive to GPX4 depletion and accompanied by regulatory cell death with features of ferroptosis (Chen et al., 2015). Further studies have shown that GPX4 knockout in adult mice die from the hippocampal neuron loss (Yoo et al., 2012). Given that the key role of GPX4 for the maintenance of neuronal function, selective deletion of GPX4 in the brain contributes to cognitive impairment and neurodegeneration (Hambright et al., 2017). Besides, selenocysteine-containing GPX4 is also foremost for the survival of interneurons as prior work has depicted mice deficient selenocysteine-associated GPX4 in fatal stage exhibits severe epileptic seizures (Ingold et al., 2018).

**FIGURE 8:**
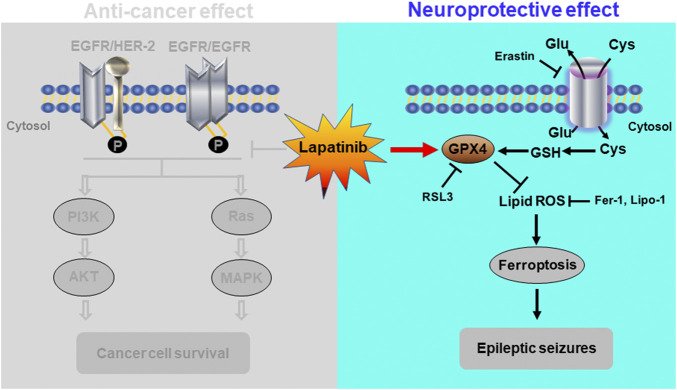
Schematic diagram depicting a novel role of suppressing neuronal ferroptosis for lapatinib in epileptic seizures. The graph not only depicted the traditional anti-cancer effect of lapatinib, but also showed the neuroprotection of lapatinib in KA-induced epileptic seizures in mice. Lapatinib protected against the KA-induced epileptic seizures by inhibiting GPX4-dependent neuronal ferroptosis.

Collectively, our present work shows that lapatinib, a well-known anti-cancer drug, protects brain against epileptic seizures. And GPX4-mediated ferroptosis was involved in the neuroprotective effect of lapatinib in epileptic seizures (Figure 8). As we all know, seizures have long been considered as a complication of AD, ischemic stroke and other neurological diseases (Camilo and Goldstein, 2004; Larner, 2010). In addition, PD, schizophrenia, and cerebral infarction appear to be primarily associated with cognitive dysfunction (Bennett et al., 2005; Green, 2006; Yang et al., 2016). So, whether lapatinib has beneficial effects on other brain diseases warrants further investigation.

## Data Availability

The original contributions presented in the study are included in the article/Supplementary Material, further inquiries can be directed to the corresponding author.

## Glossary

4-HNE: 4-hydroxynonenal;
5-LOX: 5-Lipoxygenase;
ACSL4: acyl-CoA synthetase long-chain family member 4;
AD: Alzheimer’s disease;
CNS: central nervous system;
DFO: deferoxamine;
DMEM: Dulbecco’s modified Eagle’s medium;
EEG: electroencephalogram;
Era: erastin;
FBS: fetal bovine serum;
Fer-1: ferrostatin-1;
Glu: glutamate;
GPX4: glutathione peroxidase 4;
GSH: glutathione;
HBSS: Hank’s Balanced Salt Solution;
KA: kainic acid;
Lap: lapatinib;
Lip-1: liproxstatin-1;
lipid ROS: lipid reactive oxygen species;
MDA: malonaldehyde;
NMDA: N-methyl-d-aspartate;
PD: Parkinson’s disease;
PI: propidium iodide;
PTGS2: prostaglandin endoperoxide synthase 2;
RSL3: ras-selective lethal small molecule 3;
SLC7A11: solute carrier family 7 member 11.
