# Corrigendum: Neuroprotective Effects of the Anti-Cancer Drug Lapatinib Against Epileptic Seizures *via* Suppressing Glutathione Peroxidase 4-Dependent Ferroptosis

**DOI:** 10.3389/fphar.2021.810295

**Published:** 2022-01-03

**Authors:** Ji-Ning Jia, Xi-Xi Yin, Qin Li, Qi-Wen Guan, Nan Yang, Kang-Ni Chen, Hong-Hao Zhou, Xiao-Yuan Mao

**Affiliations:** ^1^ Department of Clinical Pharmacology, Xiangya Hospital, Central South University, Changsha, China; ^2^ Institute of Clinical Pharmacology, Central South University, Hunan Key Laboratory of Pharmacogenetics, Changsha, China; ^3^ Engineering Research Center of Applied Technology of Pharmacogenomics, Ministry of Education, Changsha, China; ^4^ National Clinical Research Center for Geriatric Disorders, Changsha, China; ^5^ Department of Pediatrics, Xiangya Hospital, Central South University, Changsha, China

**Keywords:** lapatinib, epileptic seizures, neuroprotection, ferroptosis, lipid peroxidation, glutathione peroxidase 4

In the original article, there was a mistake in Figure 5F and Figure 6B as published. Due to our carelessness in the process of rearranging these figures, the image in the “Era + Fer-1” group within Figure 5F and the image in the “Lap” group within Figure 6B were uploaded with mistakes. The corrected Figure 5 and Figure 6 appears below.

**FIGURE 5:**
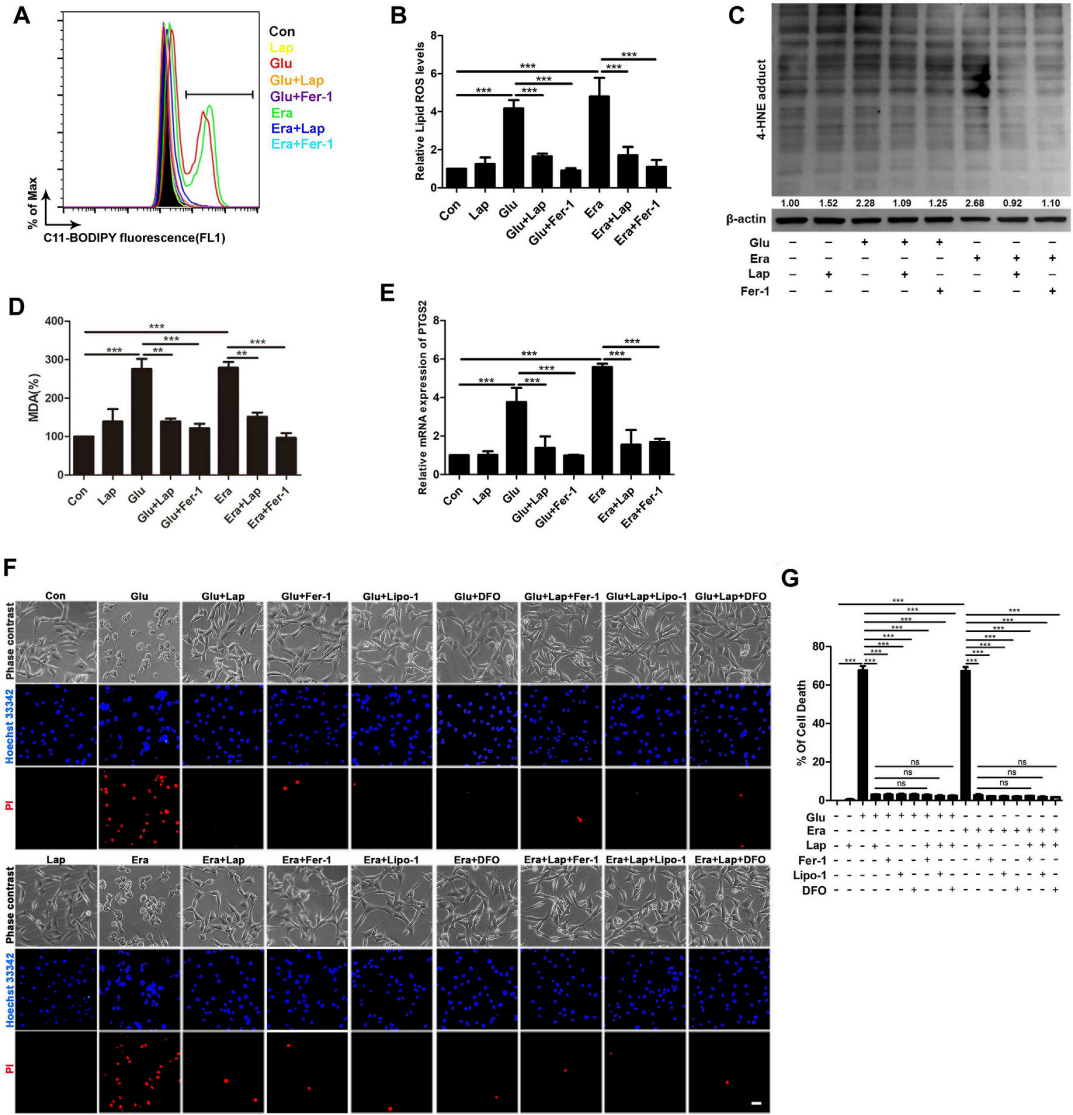
Lapatinib prevents Glu- or erastin-induced neuronal death possibly by suppressing ferroptosis **(A–D)** Detection of lipid ROS, 4-HNE and MDA content in the glutamate (Glu)- or erastin (Era)-induced HT22 cell death model following lapatinib (Lap) (10 μM) and ferrostatin-1 (Fer-1) (12.5 μM) pretreatment for 2 h **(E)** RT-qPCR analysis of PTGS2 mRNA expression pretreated with or without Lap (10 μM) and Fer-1 (12.5 μM) in HT22 cells induced by Glu or Era **(F,G)** Comparisons of combination of Lap and ferroptosis inhibitors and Lap alone in HT22 cells following Glu or Era challenge when pretreatment with Lap (10 μM), Fer-1 (12.5 μM), liproxstatin-1 (Lip-1) (1 μM) and deferoxamine (DFO) (50 μM) pretreatment for 2 h. Scale bar: 200 μm. All results were presented as the mean ± SEM from three independent experiments, ns indicates no statistical significance. **p < 0.01 and ***p < 0.001.

**FIGURE 6:**
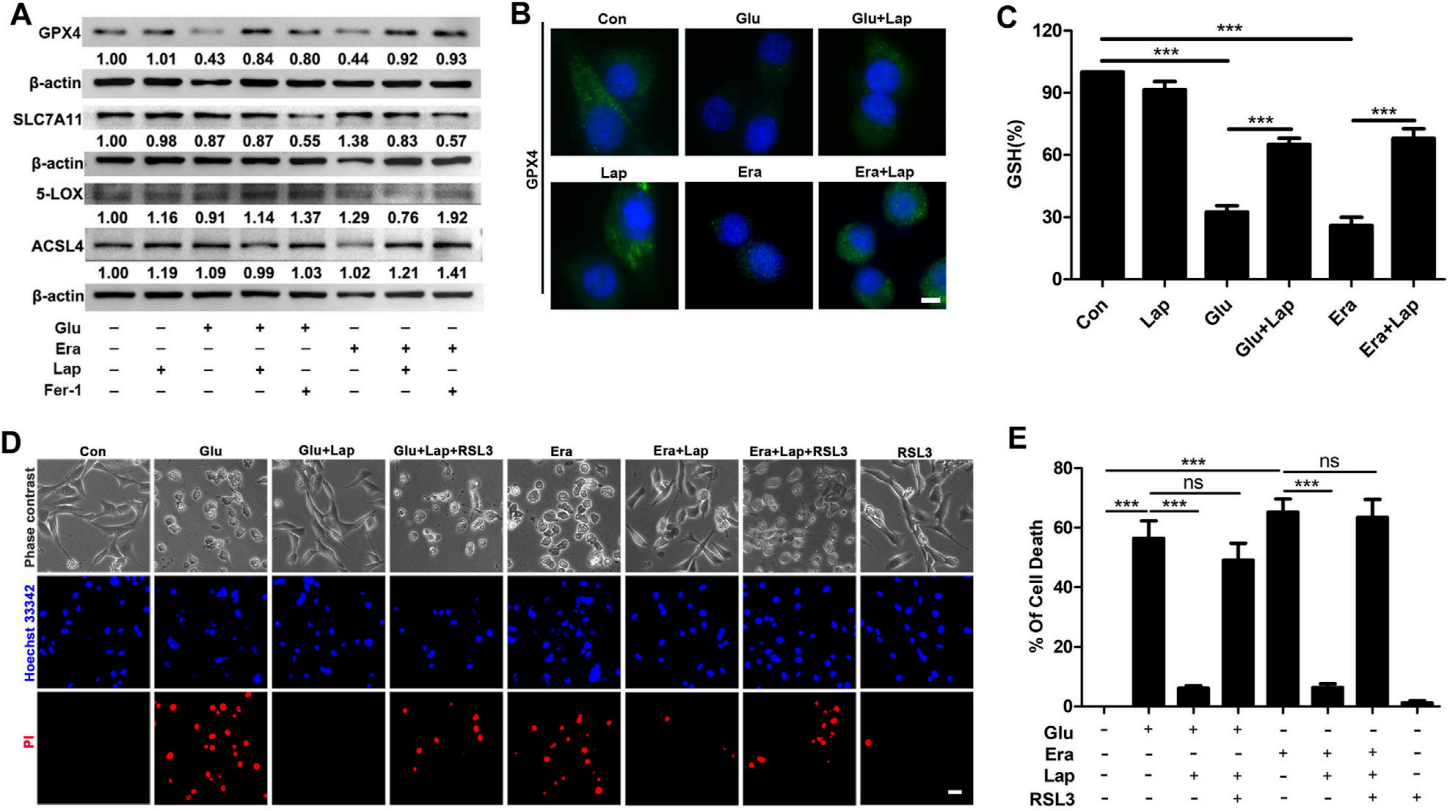
Lapatinib inhibits neuronal ferroptosis by blocking the downregulation of GPX4 **(A)** Effects of lapatinib (Lap) or ferrostatin-1 (Fer-1) on the expressions of ferroptosis-related proteins including glutathione peroxidase 4 (GPX4), solute carrier family 7 member 11 (SLC7A11), 5-Lipoxygenase (5-LOX) or acyl-CoA synthetase 4 (ACSL4) in HT22 cell death model induced by glutamate (Glu) or erastin (Era). The value below the band is obtained via ImageJ analysis **(B)** Immunofluorescence analysis of GPX4 expression after pretreatment for 2 h with Lap (10 μM), followed by exposure to Glu or Era for another 8 h in HT22 cells. Scale bar: 50 μm **(C)** GSH level was assessed following Lap (10 μM) pretreatment for 2 h **(D,E)** Effects of GPX4 inhibition by ras-selective lethal small molecule 3 (RSL3) (1 nM) on the neuroprotection of Lap (10 μM) against Glu- or erastin-induced cell death in HT22 cells. Scale bar: 200 μm. All results were shown as the mean ± SEM from three independent experiments, ns means the difference is not statistically significant, ***p < 0.001.

The authors apologize for this error and state that this does not change the scientific conclusions of the article in any way. The original article has been updated.

